# Ecotoxicological effect of imidacloprid on spore germination of phylogenetically distinct arbuscular mycorrhizal fungi species

**DOI:** 10.1007/s00572-026-01263-6

**Published:** 2026-04-28

**Authors:** Taynara Gomes Pires, Luís Carlos Iuñes de Oliveira Filho, Aline de Liz Ronsani, Osmar Klauberg-Filho

**Affiliations:** 1 Department of Soils and Natural Resources, Universidade do Estado de Santa Catarina (UDESC Lages), Lages, Santa Catarina Brazil; 2 Universidade Federal de Santa Catarina (UFSC), Florianópolis, Santa Catarina Brazil; 3 Centre for functional ecology, Department of Life Sciences, University of Coimbra, Coimbra, Portugal

**Keywords:** Insecticide, Neonicotinoid, *In vitro* assay, Pre-symbiotic phase, AMF-sandwich test

## Abstract

Arbuscular mycorrhizal fungi (AMF) establish a mutualistic symbiosis with most terrestrial plants. Mycorrhizal colonization begins with fungal spore germination in soil, a critical stage for symbiosis establishment, and is therefore highly susceptible. Among the compounds that may negatively affect AMF, imidacloprid has been identified as a potential stressor. In this context, the present study aimed to investigate the effects of the insecticide imidacloprid, as the active ingredient, on the pre-symbiotic phase of *Acaulospora morrowiae*, *Gigaspora margarita*, and *Rhizophagus clarus*. The substrate used for the in vitro assays was tropical artificial soil (TAS). The concentrations tested were 0, 2, 4, 8, 16, 32, 64, 128, 256, and 512 mg imidacloprid kg^–1^ of TAS for *A. morrowiae* and *G. margarita*; and 0, 1, 2, 4, 8, 16, 32, 64, 128, and 256 mg imidacloprid kg^–1^ of TAS for *R. clarus*. Imidacloprid reduced spore germination in all AMF species across all tested concentrations. However, *A. morrowiae* was the most sensitive species, with an IC_50_ of 23 mg kg^–1^. Although all AMF species evaluated in this study showed inhibition of spore germination upon exposure to imidacloprid, their responses differed among species, likely due to distinct morphological characteristics.

## Introduction

Arbuscular mycorrhizal fungi (AMF), members of the phylum Glomeromycota, constitute a monophyletic group of obligate symbionts that play a fundamental role in terrestrial ecosystems. Through their associations with plant roots, AMF significantly influence plant mineral nutrition, soil aggregation, and the stability and resilience of agricultural systems (Brundrett and Tedersoo 2018; Wu and Zou 2017). Despite sharing a common symbiotic lifestyle, AMF exhibit substantial phylogenetic diversity, which is reflected in pronounced differences in morphological, physiological, and ecological traits that regulate their life cycle and interactions with the soil environment (Chaudhary et al. 2014).

Recent phylogenetic analyses have demonstrated that many of these functional traits exhibit phylogenetic conservatism, whereby trait variation is constrained and conserved across evolutionary lineages (Koch et al. 2017; Powell et al. 2009). Characteristics such as spore size and morphology, spore wall thickness and composition, germination dynamics, extraradical mycelial development, and root colonization strategies vary systematically among orders and families within Glomeromycota. This phylogenetic structure suggests that AMF responses to environmental drivers and anthropogenic disturbances are not random but are at least partly shaped by evolutionary history (Antunes et al. 2025).

Within this context, exposure to chemical stressors in soil, including agricultural pesticides, may differentially affect AMF lineages according to their phylogenetic position. Experimental and review studies have shown that agrochemicals can disrupt both pre-symbiotic stages, such as spore germination, and symbiotic development, including mycelial growth and arbuscule formation. However, the magnitude and direction of these effects vary widely among species (Koch et al. 2017; Pagano et al. 2023), underscoring the need for analytical frameworks that explicitly integrate phylogeny when interpreting AMF responses to environmental stress.

Species from different orders within Glomeromycota illustrate contrasting evolutionary and functional strategies. *Rhizophagus clarus* (Glomerales, Glomeraceae) produces small-to-medium-sized spores with relatively thin walls and high germination rates, traits commonly associated with rapid root colonization and high ecological plasticity (Silva 2004; INVAM 2024c). In contrast, *Gigaspora margarita* (Gigasporales, Gigasporaceae) forms large, thick-walled, ornamented spores, exhibits slow germination that is strongly dependent on host-derived chemical cues, and does not produce vesicles, features characteristic of phylogenetically conserved lineages that are often less tolerant of disturbed environments (Arcidiacono et al. 2024; Hart and Reader 2002; INVAM 2024b). *Acaulospora morrowiae* (Diversisporales, Acaulosporaceae) occupies an intermediate phylogenetic and functional position, displaying variable spore morphology, multilayered spore walls, and the formation of both vesicles and functional arbuscules, reflecting a mixed strategy of soil persistence and root colonization (INVAM 2024a).

These phylogenetic and functional contrasts suggest that AMF sensitivity to environmental stressors may be closely linked to the degree of evolutionary conservatism in key traits. Nevertheless, although spore germination assays are widely employed to evaluate AMF responses to chemical contaminants, few studies have explicitly tested whether such responses are structured along phylogenetic lines (Chaudhary et al. 2022).

We used the neonicotinoid insecticide imidacloprid was used as a model chemical stressor to examine whether AMF sensitivity to pesticides is associated with phylogenetic divergence. Imidacloprid has been used in seed treatment or in row applications. Specifically, we evaluated the effects of imidacloprid on spore germination in three phylogenetically distinct AMF species, *A. morrowiae* (Diversisporales), *G. margarita* (Gigasporales), and *R. clarus* (Glomerales), exposed to increasing concentrations of the active ingredient. We hypothesized that chemical stress in soil inhibits spore germination in all three AMF species; however, phylogenetically distinct lineages would differ in their sensitivity due to evolutionary conservatism of morphofunctional traits related to germination and symbiotic establishment. Specifically, species with thinner spore walls and lower levels of protective compounds such as melanin (e.g., *A. morrowiae*) were expected to be more sensitive to insecticide exposure, whereas species with thicker walls and higher lipid reserves (e.g., *G. margarita*) were predicted to exhibit greater tolerance.

## Materials and methods

### Fungal species and mixed inoculum production

Spores of *Acaulospora morrowiae* (PRN108), *Gigaspora margarita* (SCT071A), and *Rhizophagus clarus* (RJN102A) were obtained from the International Culture Collection of Glomeromycota (CICG). The inclusion of these three species provides significant initial insights into a low-tier ecotoxicological assessment. As this study represents an initial screening (low-tier), this approach allows the establishment baseline toxicity data prior to more complex, high-throughput, or large-scale community studies (higher-tier studies).

The AMF species were propagated in pot cultures using a host plant, following the method described by Stutz and Morton (1996). The growth substrate consisted of a 1:1 (v/v) mixture of sand and soil, sterilized by two autoclaving cycles (60 min at 120 °C and 1 atm). The sterilized substrate was used to fill 1.5 L plastic pots. Seeds of *Brachiaria* sp. were sown to promote fungal multiplication. The pots were maintained under controlled environmental conditions (25 °C, 16 h light/8 h dark photoperiod) for 120 days, allowing symbiosis to establish and spores to be produced in the substrate.

After 120 days, the cultures were evaluated for spore presence, abundance, and purity. The inoculum was then collected and stored at 4 °C until further use (from August 2023 to April 2024).

### Tested substance

The tested substance was imidacloprid (active ingredient), with the chemical formula C_9_H_10_ClN_5_O_2_ (ANVISA 2019). According to the environmental profile provided by the Brazilian Institute of Environment and Renewable Natural Resources (IBAMA), the compound has an oral median lethal dose (LD_50_) in rats ranging from 410 to 440 mg kg^–1^ and is therefore classified as moderately toxic (IBAMA 2019). According to product labels registered in Brazil, imidacloprid is classified as Toxicological Category III (moderately toxic product) (Agria Brasil Ltda., 2024).

Concentrations of 0, 2, 4, 8, 16, 32, 64, 128, 256, and 512 mg kg^–1^ of tropical artificial soil (TAS) were applied to *A. morrowiae* and *G. margarita*, whereas concentrations of 0, 1, 2, 4, 8, 16, 32, 64, 128, and 256 mg kg^–1^ of TAS were applied to *R. clarus*, with five replicates per concentration. The selected concentrations were based on available literature concerning the sensitivity of each AMF species. For *A. morrowiae* and *G. margarita*, no studies reporting tolerance thresholds or toxicity levels for this insecticide were found; therefore, a broader concentration range was adopted to encompass concentrations from environmentally low levels to potentially toxic concentrations. In contrast, for *R. clarus*, sensitivity to imidacloprid has been previously documented (Malfatti et al. 2023), and a narrower concentration range consistent with previously values was applied. We selected a range of imidacloprid concentrations to ensure the determination of IC_50_ values and to establish clear dose-response relationships for different AMF species. Nominal concentrations of imidacloprid were used as reference values and applied in the statistical analysis, and actual concentrations in the substrate were not measured.

For soil contamination, imidacloprid was dissolved in acetone to prepare a stock solution, which was subsequently added to the TAS to obtain the desired concentrations. After contamination, the substrate was thoroughly homogenized and allowed to stand for 2 h to ensure complete evaporation of acetone. Both stock solution preparation and TAS contamination were performed on the same day as the establishment of the experimental units (EUs).

### Experimental procedure

Spore germination assays were conducted following the protocol described by Mallmann et al. (2018). Each experiment included three treatments: (i) Control (C0), consisting of Petri dishes containing uncontaminated TAS and inoculated with AMF; (ii) Solvent control (Ct_sol_), consisting of Petri dishes containing uncontaminated TAS supplemented with acetone used as solvent for the active ingredient, and inoculated with AMF; and (iii) Test treatments, consisting of Petri dishes containing TAS contaminated with imidacloprid at the concentrations described above and inoculated with AMF.

For each AMF isolate, spores were extracted from pure cultures using the wet sieving method (Gerdemann and Nicolson 1963), followed by centrifugation in a sucrose gradient (20% and 60%). Only spores with intact walls were selected, while empty or damaged spores were discarded. The selected spores were placed on filter paper slightly moistened with distilled water and subsequently transferred to nitrocellulose membranes (47 mm diameter, 0.45 μm pore size, white, gridded with a 3 mm mesh), previously moistened with distilled water. For each experimental unit, 30 spores were selected and randomly distributed on a nitrocellulose membrane. For each concentration tested, five replications (*n* = 5) were prepared, totaling 42 experimental units for each fungal species tested.

The membrane containing the spores was covered with a second nitrocellulose membrane, also moistened with distilled water, forming a “spore sandwich,” which was then placed between two layers of TAS in Petri dishes. Each experimental unit consisted of a Petri dish containing an initial layer of substrate (40 g), onto which the spore sandwich was placed, followed by a second layer (40 g) of the same substrate.

Petri dishes were sealed with plastic film and incubated in B.O.D. (Biochemical Oxygen Demand) type germination chambers at 28 °C (Mallmann et al. 2018), in complete darkness for 14 days. After the incubation period, the experimental units were opened, and the spore sandwiches were removed from the Petri dishes. The membranes were then separated, and spore germination was evaluated under a stereomicroscope.

A spore was considered germinated when the length of the germ tube was at least five times the spore diameter. The total number of recovered spores and the number of germinated spores were quantified for each species and replicate. Germination percentage was calculated as (*x* × 100)/n, where *x* is the number of germinated spores and *n* is the number of recovered spores. Results were considered valid when they met the criteria established by the ISO 10,832 protocol (ISO 2009), as follows: (i) the mean number of recovered spores was equal to or greater than 25, and (ii) the mean spore germination percentage in the control treatment was equal to or greater than 75%.

### Statistical analysis

Spore germination data were first subjected to tests for normality (Shapiro-Wilk test) and homogeneity of variances (Bartlett’s test). A preliminary t-test was conducted between the solvent control (Ct_sol_) and the water control (C0) to verify whether significant differences in spore germination occurred. The Ct_sol_ was used as the reference control for statistical comparisons. Data were analyzed using one-way analysis of variance (ANOVA), followed by Dunnett’s test (*p* < 0.05) to evaluate differences between each tested concentration and the CT_sol_. This approach allowed determination of the no-observed-effect concentration (NOEC) and the lowest-observed-effect concentration (LOEC).

Based on germination percentage data, the effective inhibition concentration for 50% of the population (IC_50_) and the corresponding 95% confidence intervals were estimated using nonlinear regression models, following the guidelines of Environment Canada (EC 2005). Model fitting was performed using the Levenberg-Marquardt algorithm, with the model that best fit the observed data selected.

## Results

**Fig. 1:**
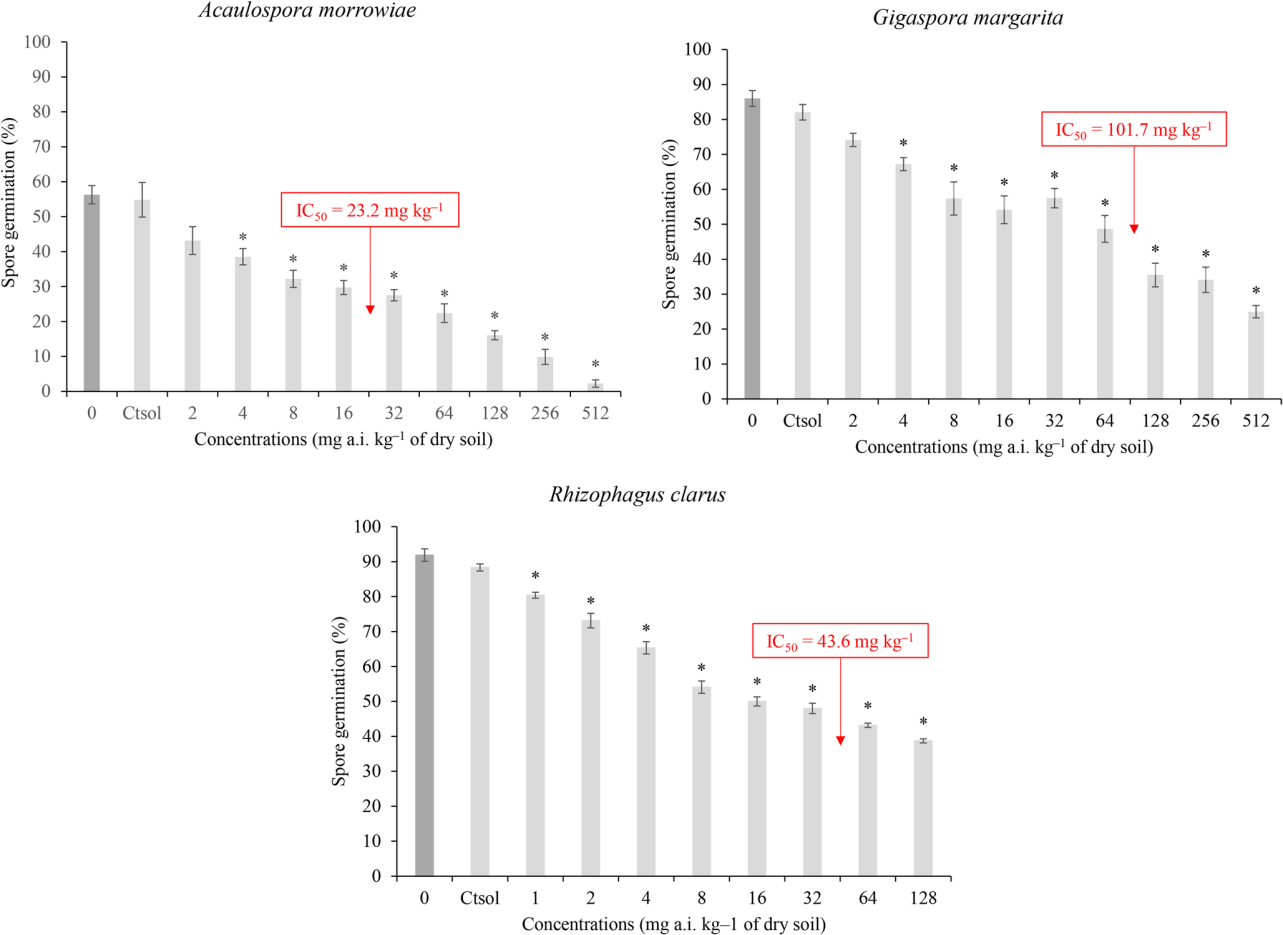
Spore germination (%) of *Acaulospora morrowiae*, *Gigaspora margarita*, and *Rhizophagus clarus* in TAS contaminated with different concentrations of imidacloprid (active ingredient, a.i.). Asterisks indicate statistically significant differences compared with the control, based on Dunnett’s test (*p* < 0.05). Data are presented as mean ± SD. The dark gray bar indicates spore germination in uncontaminated TAS (distilled water); the solvent control (Ct_sol_) was used as the reference control

In all spore germination assays, the mean number of spores recovered per experimental unit was greater than 25, thus meeting the assay validation criteria proposed by Mallmann et al. (2018). No significant differences (*p* > 0.05) were observed between the Ct_sol_ and C0 of any species, confirming that acetone did not affect spore germination. A significant effect of imidacloprid was observed for all species, as indicated by reduced spore germination percentages (Fig. 1).

In assays with *A. morrowiae* and *G. margarita*, significant reductions in germination percentage were observed at 4 mg kg^–1^ (*p* < 0.05), with decreases of of 29.7% and 18.1%, respectively, compared with the control. The NOEC was 2 mg kg^–1^, and the LOEC was 4 mg kg^–1^. According to the fitted logistic model, the IC_50_ was 23.2 mg kg^–1^ (95% CI: 12.5–33.8) for *A. morrowiae* and 101.7 mg kg^–1^ (95% CI: 57.1–146.2) for *G. margarita*. At 2 mg kg^–1^, germination inhibition reached 21.3% and 9.6% for *A. morrowiae* and *G. margarita*, respectively, increasing to 95.9% and 69.6% at the highest concentration tested (Table 1).

**Table 1:** Germination inhibition (%) of *Acaulospora morrowiae*, *Gigaspora margarita*, and *Rhizophagus clarus* following exposure to imidacloprid

	Concentrations (mg kg^–1^)									
	Ctsol	2	4	8	16	32	64	128	256	512
*A. morrowiae*	2.5	21.3	29.7	41.2	45.8	49.8	59.2	70.7	82.0	95.9
*G. margarita*	4.6	9.6	18.1	30.1	34.0	29.9	40.7	56.7	58.4	69.6
	Ctsol	1	2	4	8	16	32	64	128	
*R. clarus*	3.9	8.9	17.2	26.1	38.8	43.4	45.6	51.1	56.1	

In the assay with *R. clarus*, a significant reduction in germination (8.9%) was observed at 1 mg kg^–1^ (*p* < 0.05) compared with the control, indicating that the LOEC corresponded to the lowest concentration tested. According to the fitted logistic model, the IC_50_ was 43.6 mg kg^–1^ (95% IC: 30.3–57.1). Germination inhibition was 8.9% at 1 mg kg^–1^ and reached 56.1% at the highest concentration tested (Table 1).

## Discussion

We focus on spore germination, the most critical phase of the AMF life cycle for the establishment of mycorrhizal associations. While subsequent stages, such as root colonization, are vital for the functional outcomes of the symbiosis, spore germination serves as a primary ecological filter (low-tier assessment). Imidacloprid appears to act at this early stage of the AMF life cycle, potentially reducing viable spore density in agricultural soils. This effect may be particularly critical because it compromises the establishment of symbiosis. The active ingredient imidacloprid negatively affected spore germination in all evaluated AMF species, corroborating the hypothesis, which predicted that spore germination of *A. morrowiae*, *G. margarita*, and *R. clarus* would be reduced by imidacloprid (a.i.). *A. morrowiae* did not reach the minimum germination percentage established by the ISO 10,832 protocol for *Funneliformis mosseae* (ISO 2009). Germination rates can vary considerably depending on both experimental conditions and the AMF species, often reflecting species-specific responses as reported by Mallmann et al. (2018). Furthermore, the genus *Acaulospora* has been reported to exhibit some quiescence behavior, which may influence the “minimum germination” required, something that still needs to be better defined considering phylogenetics traits (Tommerup 1983). Because the present study did not assess the viability of non-germinated spores, it remains uncertain whether ungerminated propagules were non-viable or remained viable but dormant. For future studies, it would be appropriate to include an analysis of the viability of non-germinated spores using vital dyes, in order to distinguish dead spores from those that remain viable but in a state of quiescence or induced dormancy. However, we note that the current experimental protocol does not include this type of evaluation, and its feasibility within the methodological framework we adopted still needs to be carefully assessed and tested in terms of applicability. This observation reinforces the need to adjust experimental conditions to accommodate the biological particularities of each arbuscular mycorrhizal fungus (Mallmann et al. 2018). Therefore, *A. morrowiae* was retained for subsequent assays.

The IC_50_ values reported here indicate pronounced interspecific differences in sensitivity to imidacloprid among the evaluated AMF taxa. *A. morrowiae* exhibited the highest sensitivity, whereas *G. margarita* was the least sensitive species, supporting the hypothesis that sensitivity to imidacloprid varies among phylogenetically distinct AMF species. Similar interspecific variability in AMF responses to agrochemicals has been reported previously (Malfatti et al. 2023; Mallmann et al. 2018). Notably, Malfatti et al. (2023) demonstrated that *R. clarus* was more sensitive to the imidacloprid-based commercial formulation Much 600 FS^®^ than *G. albida*, reinforcing the view that responses to neonicotinoids are strongly species dependent. A comparable pattern was reported by Mallmann et al. (2018) for the organophosphate insecticide chlorpyriphos: *R. clarus* exhibited pronounced reductions in spore germination and extraradical mycelial development, whereas *G. albida* showed greater tolerance, with only minor or non-significant effects on these parameters. Together, these studies indicate that AMF responses to insecticides are shaped not only by the chemical properties of the compounds but also by intrinsic species-specific sensitivities and their traits. Consistent with this interpretation, at the highest tested concentration (512 mg kg^–1^ of imidacloprid), *G. margarita* still exhibited 25% spore germination, indicating higher tolerance than *R. clarus* and *A. morrowiae.* According to Koch et al. (2017), such interspecific differences may be linked to variation in spore morphology, as structural traits, including thicker spore walls, multiple differentiated wall layers, and differences in lipid matrix composition, may enhance resistance by limiting the penetration of toxic molecules. Despite the fact that morphological and physiological traits such as wall thickness or lipid reserves have been proposed in the literature as possible factors influencing sensitivity, our study did not generate direct data to support these mechanisms. Nevertheless, these represent plausible hypotheses that could be addressed through targeted analyses, such as spore wall composition or metabolic profiling, to better understand the variability in sensitivity among genera.

Overall, the differential responses of AMF species to imidacloprid are likely linked to morphological, physiological, and ecological traits (Hage-Ahmed et al. 2019; Rivera-Becerril et al. 2017). Spores with thicker walls or higher concentrations of hydrophobic compounds, including lipids and melanin, tend to exhibit increased resistance to insecticide penetration (Koch et al. 2017). In addition, interspecific differences in antioxidant enzyme activity and detoxification mechanisms play a crucial role in tolerance to chemical stress (Hage-Ahmed et al. 2019; Rivera-Becerril et al. 2017). Species adapted to anthropogenically influenced environments may have developed higher tolerance levels, whereas others, such as *A. morrowiae*, appear to be more sensitive (Rivera-Becerril et al. 2017). Thus, the contrasting responses observed among *A. morrowiae*, *R. clarus*, and *G. margarita* reflect a combination of structural and physiological factors that determine species-specific sensitivity to imidacloprid. Species of Gigasporales, including *G. margarita*, are commonly associated with drier, oligotrophic, or otherwise stressful environments such as tropical dry forests (Assis et al. 2018). Their large, thick-walled spores favor resistance to soil stresses such as extreme pH, aluminum toxicity, and drought. *G. margarita* is also frequently reported in agroecosystems and tropical forests, indicating broad ecological amplitude and tolerance to moderate disturbance (Assis et al. 2018; Sylvia and Schenck 1983; Yakasai and Rabiu 2025). In contrast, many Diversisporales, including *Acaulospora*, tend to occur in soils with lower pH and are often linked to nutrient-poor or environmentally stressful conditions (Chagnon et al. 2013; Siqueira et al. 2014). Spore germination studies further show that Diversisporales often respond negatively to herbicides and fungicides, suggesting lower physiological robustness to xenobiotic compounds (Malfatti et al. 2021; Mallmann et al. 2018). This combination of life-history traits, spore morphology, and ecological niches provides a coherent framework to interpret why *A. morrowiae* emerged as the most sensitive species under imidacloprid exposure, while *G. margarita* maintained greater tolerance.

Although AMF are non-target organisms of insecticides, the present results confirm that they can be significantly affected by these compounds, highlighting the importance of investigating not only the effects of fungicides but also those of insecticides across the diverse phylogenetic lineages of this key group of soil fungi. This impact is likely due to interactions between insecticide molecules and soil microbiota, which may alter physicochemical and biological properties essential for fungal development (Hage-Ahmed et al. 2019). Moreover, neonicotinoid residues can accumulate in microenvironments surrounding spores and hyphae, interfering with key physiological processes such as cellular respiration, ion transport, and membrane integrity (Goulson 2013; Hage-Ahmed et al. 2019). However, we did not directly examine the specific physiological pathways through which these compounds influence AMF germination, which remain poorly understood.

Disruption of spore germination has direct consequences for the establishment of mycorrhizal symbiosis, as germination represents the initial step in host recognition and root colonization (Bécard and Fortin 1988; Giovannetti et al. 1999; Smith and Read 2008). Reduced germination therefore compromises symbiosis formation and, consequently, plant nutrient acquisition (Bago et al. 2020; Smith and Read 2008). This impairment can negatively affect soil nutrient dynamics, decrease phosphorus and nitrogen use efficiency, and weaken soil aggregate formation and stability (Rillig and Mummey 2006; van der Heijden et al. 2015). As a result, insecticide-induced effects on AMF may ultimately reduce plant productivity and compromise ecological functioning in agricultural soils (Hage-Ahmed et al. 2019; Rivera-Becerril et al. 2017). In the 1990s, Nancy Collins Johnson and colleagues demonstrated that crop management practices in the American Corn Belt can exert strong selective pressures on AMF communities, with lasting consequences for crop productivity. Based on long-term field experiments in Minnesota, their studies showed that continuous fertilization and monoculture systems favored the dominance of AMF taxa that were less beneficial, or even detrimental, to host plant performance, contributing to yield declines in continuous corn and soybean systems (Johnson et al. 1992). Importantly, these shifts were not random but reflected consistent changes in community composition, suggesting that management practices act as environmental filters that select for specific functional strategies within the AMF phylogeny. In this context, the observed prevalence of “less cooperative” fungi supports the idea that key functional traits, such as carbon allocation patterns and growth strategies, may exhibit phylogenetic conservatism. These findings underscore the need to consider evolutionary history when evaluating how additional anthropogenic pressures further shape AMF community structure and function across species and phylogenetic lineages (Chagnon et al. 2013), including the additional influence of agrochemical inputs. Based on the study by Chagnon et al. (2013), species traits largely define functional roles, and taxa occurring in more conservative systems from an anthropogenic perspective, as well as in natural soils, may exhibit greater tolerance to environmental stresses. However, despite this ecological resilience, these species are not necessarily tolerant to xenobiotics such as agrochemicals.

Previous studies have already documented the effects of agrochemicals on AMF spore germination (Lunardi et al. 2025; Malfatti et al. 2023; Rivera-Becerril et al. 2017). Malfatti et al. (2023) reported species-specific responses of AMF to thiamethoxam and imidacloprid, consistent with the present findings, in which *A. morrowiae*, *G. margarita*, and *R. clarus* exhibited distinct sensitivities to the same active ingredient.

While the higher concentrations tested may exceed average field-realistic levels typically reported in the literature (which often range from 0.012 to 0.018 mg kg^–1^, with values of up to 0.65 mg kg^–1^ reported; Anderson and Harmon-Threatt 2019), they serve as a critical reference for worst-case environmental scenarios. Such high-exposure conditions can occur in agricultural hotspots, including soil surface layers immediately after application or in regions with intensive pesticide cycles. Furthermore, establishing these thresholds is a prerequisite for low-tier ecotoxicological screenings, providing a benchmark against which future high-tier, field-based studies can be compared. Although average environmental concentrations are lower, the existence of contamination hotspots and the potential for accumulation over successive cultivation cycles make the determination of acute toxicity thresholds essential for establishing safety limits and for assessing the ecological risks to soil microbial communities. These concentrations simulate hotspots that occur due to accumulation, especially given that imidacloprid’s half-life in soil can range from 174 up to 578 days (PPDB 2026). Long half-lives increase the potential for imidacloprid to accumulate in the soil (Didović et al. 2022), making this worst-case scenario possible in the field.

Finally, although AMF are non-target organisms, the results clearly demonstrate that imidacloprid can significantly impair their viability and functionality in distinct ways across different phylogenetic lineages and their representative species. Given the central role of AMF in nutrient cycling and agricultural sustainability, further research should address additional life-cycle stages and environmental conditions, including host-associated systems and multi-stage life cycle assessments, to fully quantify long-term ecological consequences (higher-tier studies).

## Conclusion

The species evaluated in this study showed inhibition of spore germination upon exposure to the active ingredient imidacloprid. However, *A. morrowiae* was more sensitive than *G. margarita* and *R. clarus*. The variation in sensitivity among species and genera highlights the need to expand research by incorporating a broader diversity of species and their phylogenetic variability. Currently available knowledge remains limited, which hinders precise conclusions regarding the actual effects of imidacloprid on these organisms.

Considering the ecological relevance of AMF, it is essential to further investigate the potential risks affecting these fungi, as their presence is fundamental to the survival of many other species. Regarding the experiment setup, while the use of in vitro conditions and standardized substrates (TAS) provides high levels of experimental control and reproducibility, these settings do not fully replicate the complex interactions of natural soil ecosystems. In field conditions, factors such as organic matter, clay, pH, and the presence of microbial communities may modify the toxic effects observed. Nonetheless, our approach was designed as a low-tier ecotoxicological screening, representing a “worst-case scenario” (high bioavailability), and constitutes a fundamental step for developing more complex, higher-tier assessments that include soil-plant-insecticide interactions.

## Data Availability

No datasets were generated or analysed during the current study.
